# Aortic dissection false lumen treatment with novel shape memory polymer devices

**DOI:** 10.1016/j.jvscit.2026.102326

**Published:** 2026-05-18

**Authors:** Dai Yamanouchi, Yusuke Sakurai, Masaru Nemoto, Sohei Matsuura, Ryohei Maeno

**Affiliations:** a Division of Vascular Surgery, Department of Surgery, University of Wisconsin, Madison, WI; b Department of Vascular Surgery, Fujita Health University, Toyoake, Aichi, Japan

**Keywords:** Aneurysm, Aortic dissection, False lumen embolization, Shape memory polymer, Thoracic stent graft

## Abstract

In this case series, innovative shape memory polymer (SMP) devices were used to treat the aortic dissection false lumen (FL) of five patients. The open-cell, porous structure of expanded SMP contributes to its radiolucency, low radial force, and conformability. The devices were designed to address the unique needs of treating the FL: sufficiently large volume, detachable and stable to minimize migration risk, segmented for flexibility, and with a range of dimensions. All five cases were technically successful. A decrease in total aortic diameter, true lumen restoration, and FL reduction were observed through 6- to 12-month follow-up. The mean changes in maximum aorta diameter from preoperative measurements were −5.8 ± 6.9 mm (30 days, n = 5), −12.0 ± 9.4 mm (6 months, n = 4), and −9.3 ± 0.8 mm (12 months, n = 2). No SMP device/procedure-related complications occurred.

Aortic dissection false lumen (FL) treatment options are categorized as FL exclusion and embolization. FL exclusion methods include the candy-plug technique, physician-modified aortic occluders, the knickerbocker technique, and the cork-in-the-bottle technique.^1^ FL embolization is performed using a variety of off-label peripheral vascular embolization devices.^2^, ^3^, ^4^, ^5^ Shape memory polymer (SMP) is a novel radiolucent material that self-expands to a porous scaffold on delivery into a vessel and is designed to support thrombus formation throughout its structure. The objective of this case series was to determine the safety and efficacy of SMP devices specifically designed to meet the needs of FL treatment after aortic dissection.

## Methods

**Fig 1:**
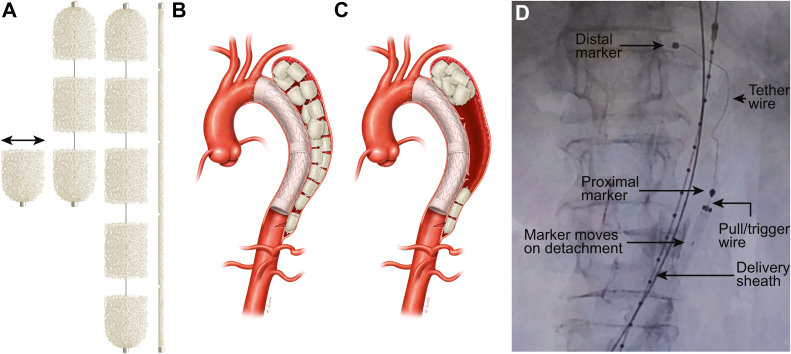
**(A)** Shape memory polymer (SMP) devices (Shape Memory Medical), available with one, three, and five polyurethane SMP segments and 18-mm, 24-mm, and 36-mm diameter of the expanded SMP segments (*arrow*). The SMP segments in multisegment devices are tethered via a platinum/iridium wire. The devices are detachable via a mechanical twist-and-release mechanism (Supplementary Video, online only). The SMP segments are crimped for catheter delivery (five-segment device example, right) and self-expand on delivery into a vessel. **(B)** Illustration of multiple tethered SMP devices deployed into the false lumen (FL), including devices laying parallel to one another. **(C)** Illustration of partially filling an FL with SMP devices—proximally, and in the location normally occupied by a candy plug. **(D)** Intraprocedural image showing the terminal platinum/iridium marker bands of a three-segment, 18-mm-diameter device and detachment mechanism features.

The SMP devices (Fig 1, *A*; Shape Memory Medical) were not approved in Japan at the time of treatment and were obtained through the physician-directed personal import framework for unapproved medical devices,^6^ under which a physician may directly import a device for use in the physician's own patients under the physician's responsibility, subject to applicable import confirmation procedures; institutional approval was obtained before use, and written informed consent for treatment and publication were obtained from all patients. At the time of submission for publication, the SMP devices (Fig 1, *A*) were not available in any region for any indication. A patent FL and an aorta >55 mm diameter and/or a rapidly expanding aorta (≥5 mm within 6 months before the procedure) were criteria for treatment.^7^ Patients were treated and followed per the hospital standard of care for aortic dissection, with computed tomography angiography (CTA) studies at discharge, 30 days, 6 months, and 12 months postprocedure (as available to date). Multiphasic contrast-enhanced CTA parameters were nonenhanced with a slice width and interval of 1 mm, arterial phase, and delayed phase (maximum 300 seconds).

Technical success was defined as successful placement of the SMP devices at the intended target site with successful retrieval of the delivery catheter. Efficacy outcomes were determined over the zones treated with SMP devices (identified by the radiopaque makers) and are reported as change in maximum aorta diameter, FL perfusion volume, and the true lumen (TL)-to-aorta and FL-to-aorta cross-sectional area ratios, measured at the maximum aorta diameter. The arterial phase, axial slices of the CT were used for all efficacy outcome measurements; the delayed and noncontrast phases were used for reference if needed. Contouring of the TL was performed over the zones treated with SMP devices (TeraRecon), and then contouring of the outer aorta wall was performed over the same region. TL and aorta cross-sectional areas were calculated at the maximum aorta diameter in the defined region. The FL cross-sectional area was calculated as aorta minus TL and therefore included the residual flow in addition to any thrombosed regions. The TeraRecon “Region Tool” was used to select all contrast-enhanced voxels within the FL over the same defined region; FL perfusion volume was the sum of the selected voxels.

## Innovative technique

The SMP devices are available with one, three, or five tethered segments of SMP as 18-, 24-, and 36-mm-diameter devices (Fig 1, *A*). The SMP is crimped for sheath delivery (minimum ID 10-16F) and self-expands on delivery into a vessel. The detachable SMP devices allow targeted placement to either fill the FL (Fig 1, *B*) or treat specific locations such as a voluminous proximal region and/or a narrow distal channel (Fig 1, *C*).

Preoperative CTA analysis was used to determine a preliminary target region and device selection, based on access options, FL dimensions, and branch vessel management. With access, the sheath tip is positioned at the most proximal end of the target region, and the device is advanced until the distal segment just exits the sheath. The sheath is slowly retracted to unsheath the implant into the FL while maintaining gentle forward pressure to deliver in the planned location (Supplementary Video, online only). Initial expansion takes approximately 3 minutes; the SMP segments are initially stiff but quickly soften as the SMP expands, and repositioning is possible at this time. Full SMP expansion is expected in approximately 10 minutes. The first device is prone to the most movement because of surrounding untreated space; subsequent devices are less likely to move with other devices in the region. Detachment is via a simple and stable twist-and-release mechanical mechanism (Supplementary Video, online only); the radiopaque marker on the pull/trigger wire moves under fluoroscopy as detachment occurs (Fig 1, *D*).

## Results

**Fig 2:**
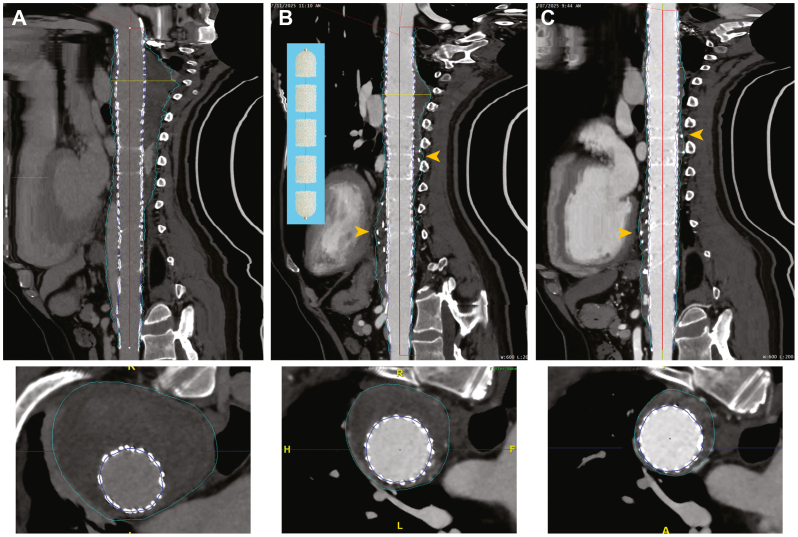
Case example (case 4). **(A)** Preoperative. A subacute patient presented with a false lumen (FL) extending from zone 2 through zone 8 after an initial intervention to treat the primary tear and thoracic region 3 weeks before implantation with shape memory polymer (SMP) devices. Aorta diameter 66.5 mm, FL perfusion 92.8 mL, 17.8% true lumen (TL)/aorta cross-sectional area ratio, 82.2% FL/aorta cross-sectional area ratio. **(B)** Thirty-day follow-up after five-segment, 24-mm diameter SMP devices (inset) were implanted from zone 3 through zone 5; a total SMP volume of 225 mL, assuming full expansion. No concomitant procedures occurred. Aorta diameter 48.7 mm, FL perfusion 2.2 mL, TL/aorta cross-sectional area ratio 39.9%, and FL/aorta cross-sectional area ratio 60.1%. The *yellow arrows* indicate radiopaque features of the SMP device (terminal marker bands, tether wire). **(C)** Six-month follow-up. Aorta diameter 40.6 mm, FL perfusion 2.0 mL, TL/aorta cross-sectional area ratio 59.9%, and FL/aorta cross-sectional area ratio 40.1%. TL/aorta and FL/aorta cross-sectional area ratios were measured at the maximum aorta diameter; all measurements are over the SMP-treated zones. The *yellow arrows* indicate radiopaque features of the SMP devices (terminal marker bands, tether wire).

Five patients were treated between August 2024 and July 2025. Patients presented with a range of dissection conditions and other aortic pathologies expected in clinical practice (Table I). Four patients had prior interventions for dissection, and one patient was treatment naïve. Technical success was 100%; multiple SMP devices were placed in the FL (Table I). The volume of implanted SMP was 123-474 mL, based on the fully-expanded volume. Overall, SMP devices were implanted from zone 3 to zone 9 (Table I, Fig 2). In our experience, it was possible to confidently place the SMP devices in patients with multiple (large) tears without migration/protrusion into the TL (eg, cases 4 and 5). In contrast to a candy plug, the low radial force of the SMP enabled FL treatment without opposing TL support (eg, cases 1, 3, and 5), which reduced the TL device burden. Concomitant TL procedures occurred in all except case 4 (Table I).

**Table I:** Baseline characteristics, prior aortic dissection treatment, and procedural details

Case	1	2	3	4	5
Age, years	71	72	60	54	72
Chronicity^a^	Chronic	Chronic	Chronic	Subacute	Chronic
False lumen, index^b^					
Primary entry tear	4	2/3	0	4	2
Proximal extension, zone	3	3	0	3	2
Distal extension, zone	10 R	11 R	11 L, R	8	9
False lumen, presenting^b^					
Proximal extension, zone	5	2/3	0	2	1
Distal extension, zone	10	11 R	11 L, R	8	9
Other aortic pathology	–	Arch aneurysm	–	–	AAA
Prior dissection intervention	TEVAR	None	FET	TEVAR PETTICOAT	TEVAR
Time since last intervention	7 months	–	12 months	3 weeks	4 months
Indication for intervention	Rapid expansion	Rapid expansion >55-mm aorta	Rapid expansion (d-SINE) >55-mm aorta	>55-mm aorta	>55-mm aorta (abdominal)
Procedural details					
Treated false lumen zone range^c^	5-9	3-6	5-9	3-5	3-9
SMP devices^d^ implanted, No	18 × 3, 1 36 × 3, 1 36 × 1, 3^e^	36 × 5, 5	36 × 5, 3	24 × 5, 5	36 × 5, 3 36 × 3, 2 18 × 3, 1
SMP volume, mL^f^	123	474	284	225	407
Concomitant procedures/devices	Aortic cuff	TEVAR PETTICOAT SCA coil embolization	TEVAR extension PETTICOAT Aortic cuff Renal stent graft	–	EVAR

*AAA*, Abdominal aortic aneurysm; *d-SINE*, distal stent graft-induced new entry; *EVAR*, endovascular aneurysm repair; *FET*, frozen elephant trunk; *PETTICOAT*, provisional extension to induce complete attachment; *SCA*, subclavian artery; *SMP*, shape memory polymer; *TEVAR*, thoracic endovascular aortic repair.

a Time from symptom onset: chronic >90 days; subacute, 15-90 days, per aortic dissection reporting standards.^6^

b Zone 0-11 definitions per aortic dissection reporting standards.^6^

c SMP device marker (most proximal) to marker (most distal).

d Listed as SMP device diameter (mm) × number of SMP segments. Refer to Fig 1 for device configurations.

e Patient was also implanted with 3 × IMPEDE-FX Embolization Plugs (12-mm-diameter pushable SMP devices, indicated for peripheral embolization), volume: 3 × 1.25 mL = 3.75 mL to embolize patent feeder vessels and false lumen.

f Total SMP volume assuming full expansion.

All patients showed a decrease in maximum aorta diameter from preoperative measurements through available follow-up (Table II): mean changes ± standard deviation were −5.8 ± 6.9 mm (30 days), −12.0 ± 9.4 mm (6 months), and −9.3 ± 0.8 mm (12 months). Mean FL flow reductions were 94.9% (30 days), 97.4% (6 months), and 97.3% (12 months). TL restoration was up to 60% of the aorta cross-sectional area with a corresponding FL reduction (Table II). The subacute case showed a notably larger 6-month decrease in maximum aorta diameter than the chronic cases (−25.9 mm vs mean ± standard deviation of −7.3 ± 2.0 mm). No device- or procedure-related complications occurred, and no evidence of malperfusion or postimplantation syndrome was observed during follow-up.

**Table II:** Aorta and lumen size outcomes (over the zones treated with SMP devices) and reinterventions

Case^a^	1	2	3	4	5
Maximum aorta diameter, mm					
Preoperative	46.0	62.2	52.4	66.5	43.1
Discharge	41.8	65.3	51.5	53.1	41.8
30 days	41.8	61.3	48.4	48.7	41.2
6 months	36.6	–	44.9	40.6	37.8
12 months	36.1	–	43.6	–	–
Change^b^	−9.9	−0.9	−8.8	−25.9	−5.2
False lumen perfusion, mL					
Preoperative	61.6	377.6	170.1	92.8	88.4
Discharge	1.6	80.2	15.0	4.6	25.3
30 days	0.9	0.3	13.1	2.2	12.3
6 months	0.3	–	NR^c^	2.0	4.7
12 months	0	–	9.2	–	–
True lumen cross-sectional area/aorta cross-sectional area, %^d^					
Preoperative	21.1	5.5	5.6	17.8	10.6
Discharge	26.9	25.1	16.5	29.9	21.1
30 days	25.9	29.1	20.6	39.9	30.4
6 months	35.7	–	24.6	59.9	41.6
12 months	38.8	–	27.2	–	–
False lumen cross-sectional area/aorta cross-sectional area, %^d^					
Preoperative	78.9	94.5	94.4	82.2	89.4
Discharge	73.1	74.9	83.5	70.1	78.9
30 days	74.1	70.9	79.4	60.1	69.6
6 months	64.3	–	75.4	40.1	58.4
12 months	61.2	–	72.8	–	–
Reinterventions^e^	–	Type 1a EF FET^f^	–	–	–

*EF*, Entry flow; *FET*, frozen elephant trunk; *SMP*, shape memory polymer; *TEVAR*, thoracic endovascular aortic repair.

a Two patients reached 12-month follow-up, two patients reached 6-month follow-up to date, and one patient declined further follow-up after 30 days.

b Change from preoperative to last available follow-up measurement.

c Not recorded. Only noncontrast computed tomography was only available; residual flow was not discernable for this timepoint.

d Measurements at the maximum aorta diameter, except case 5, in which measurements were consistently determined at the most constrained true lumen cross-section (the distal end of the pre-existing TEVAR stent graft) as the maximum aortic diameter of the untreated region; the largest aortic diameter was in the TEVAR-treated region.

e Aortic dissection-related reinterventions through the follow-up term available for each case.

f EF was surgically repaired. FET to address a kink in the TEVAR stent graft after treatment due to constrictive patient anatomy.

## Discussion

After treatment, all cases exhibited substantial changes in all three aspects of positive aortic remodeling per reporting standards^8^ at last follow-up, namely FL reduction and no growth in total aortic diameter, TL expansion and no growth in total aortic diameter, and total aortic maximal diameter reduction. Cross-sectional area was used in preference to diameter for TL and FL measurements to accommodate ellipsoidal/crescent lumens. For context, a large candy-plug series (155 patients at baseline, 142 at follow-up) reported a preoperative median maximum aortic diameter of 61 mm (interquartile range [IQR], 38-111 mm) and 54 mm (IQR, 38-90 mm) at a median follow-up of 23 months (IQR, 6-87 months).^9^ A >5 mm decrease in aorta diameter (first postoperative to most recent scan) was achieved in 68 of 142 (47%) patients, 69 of 142 (49%) were stable, and a >5 mm increase in 5 of 142 (4%).^9^ From systematic review, FL embolization with coils/peripheral vascular plugs resulted in 153 of 198 (79.3%) patients with “complete FL thrombosis” at approximately 11 months postprocedure.^2^

In clinical studies, pushable SMP devices (1.25 mL per device, delivered five at a time) from the same manufacturer (Shape Memory Medical), and using the same SMP platform as the SMP devices in this case series, have been used to actively manage abdominal aortic aneurysms at the same time as endovascular aneurysm repair with significant sac regression through 12 months postprocedure.^10^^,^^11^ Strips of porous polyvinyl alcohol sponge have also been shown to support FL obliteration and TL restoration when inserted into the FL during surgical repair.^12^ Therefore, it is possible that porous polymer structures that allow tissue ingrowth have properties that are suited to support positive aortic remodeling. Endovascular FL treatment presents unique challenges for embolization devices in that the volume requiring treatment is large and elongated, and specific placement is needed to minimize complications such as malperfusion or migration. Aortic dissection patients also undergo repeated CTA imaging studies over the long term, and metal artifacts interfere with diagnostic clarity. The self-expanding SMP devices used in this case series have the design features to enable the targeted and stable delivery of large volumes of radiolucent embolic material into the FL, both at the same time as thoracic endovascular aortic repair and for the treatment of persistent FL perfusion after thoracic endovascular aortic repair.

The limitations of this case series are its small size and limited follow-up. Prospective studies with larger populations and longer-term follow-up are needed to further evaluate the safety and efficacy of the SMP devices and develop the FL treatment strategy and patient selection.

## Conclusions

SMP devices designed to treat the FL did not raise any safety concerns in this small case series. The remodeling results, with a decrease in overall aortic diameter, substantial TL diameter restoration, FL reduction, and minimal FL perfusion at 6 to 12 months postprocedure, support further investigation into this innovative approach to aortic dissection FL treatment.

## Funding

The shape memory polymer devices were supplied by 10.13039/100031714Shape Memory Medical.

## Disclosures

D.Y. is a consultant for Shape Memory Medical. The remaining authors report no conflicts.
